# Effect of Aqueous Extract of *Telfairia occidentalis* Leaf on the Performance and Haematological Indices of Starter Broilers

**DOI:** 10.5402/2012/726515

**Published:** 2012-03-22

**Authors:** Onu P. N.

**Affiliations:** Department of Animal Science, Ebonyi State University, PMB 053, Nigeria

## Abstract

This experiment was conducted to determine the effect of aqueous extract of *Telfairia occidentalis* (Fluted Pumpkin) leaf on the performance and haematological indices of starter broilers. A total of 200, 8-day-old broiler chicks were randomly allotted to five (5) treatments, each with 4 replicate groups containing 10 chicks and fed with standard starter broiler diets. *Telfaria occidentalis* leaves extract (FPLE) was added at 0, 40, 80, 120, and 160 mL/litre of drinking water. Growth performance and haematological indices were evaluated. Results showed that there was significant (*P* < 0.05) difference in weight gain, feed conversion, and protein efficiency ratios of the birds among the treatments. Birds fed 80 ml FPLE/litre of water had significantly the highest weight gain and the best feed conversion and protein efficiency ratios. There was no significant (*P* > 0.05) variations in the feed and water intakes of the birds. Results also show no significant (*P* > 0.05) difference in haematological indices of birds among the treatments. The results of this study indicate that, for enhanced weight gain and feed conversion efficiency, birds should be fed 80 mL FPLE/litre of water.

## 1. Introduction

The utilization of plant and leaf extracts in animal production has found widespread scientific and commercial acceptance as a strategy to improve the health status and performance of the animals [1–8]. Leaf extracts also have appetizing and digestion-stimulating properties and antimicrobial effect [9–12]. According to Ifon and Basir [13] leaf vegetables supply minerals, proteins, and vitamins which could complement the inadequacies of most feedstuffs. Subba et al. [14] reported that leaf protein has the potential for supplying good quality food proteins than would be obtained with cereals, legumes, and oil seeds. They are also rich in potassium, calcium, and magnesium [15]. *Telferia occidentalis *(fluted pumpkin) is a leaf vegetable that is widely cultivated in the tropics and subtropics. Chemically *T. occidentalis *leaf extract contains 21.31% crude protein, 6.41% crude fibre, 5.50 ether extract, 10.92% ash, and 3121ME (kcal/kg) [6].

 The protein from *T. occidentalis *leaves can be harvested and fed to farm animals as solution in the form of protein concentrates [16]. According to Ladeji et al. [15] *T. occidentalis *leaves contain 30.5% crude protein, 3.0% crude lipid, 87.3% crude fibre, and 8.4% total ash. The authors noted that the leaves had low level of tannic acid (4.75 + 0.50 mg/100 g DM) and oxalate (0.45 + 2.10 mg/100 g DM), but high level of phytic acid (20.5 + 2.10 mg/100 g DM).

 In view of the relative abundance of *T. occidentalis *leaf in the study area, the present study was designed to evaluate the performance and haematological characteristics of starter broilers fed different levels of *T. occidentalis *leaf extract.

## 2. Materials and Methods

 This experiment was conducted at the Poultry Unit of Animal Science Department, Ebonyi State University, Abakaliki with the approval of the Animal Ethics Committee of the University.

### 2.1. Preparation of *T. occidentalis Extract*

 One kilogram (1 kg) of freshly cut *T. occidentalis *leaves with the stalk was separated from the stem, washed with clean water to remove dirt and sand, drained, and chopped. This was then squeezed and filtered with sieve to obtain a homogenous extract of the fluted pumpkin leaves. The homogenous leaf extract was prepared at the interval of three (3) days and served to the animals fresh according to treatments.

### 2.2. Experimental Animals and Management

 A total of two hundred (200) 8-day-old Marshal Strain commercial broiler chicks were randomly allotted to five (5) treatments in a completely randomized design (CRD). Each treatment was replicated four (4) times with ten (10) birds per replicate and fed with commercial broiler starter diet. *Telfairia occidentalis *leaf extract was added at 0, 40, 80, 120, and 160 mL/litre of drinking water representing T_1_, T_2_, T_3_, T_4_, and T_5_, respectively. 

 Water and feed were provided *ad libitum *throughout the experimental period. Ventilation and heat were provided and adjusted according to standard broiler management procedures [17]. Supplementation of *Telfairia occidentalis *leaf extract and recording of water intake started on the first day of the experiment and was recorded daily. Feed intake was also recorded daily while body weight was recorded at the beginning of the study and subsequently weekly. Feed conversion and protein efficiency ratios were also calculated.

### 2.3. Blood Sampling

 Blood collection was carried out at the 4th week of the experiment. Four (4) birds were selected at random from each of the treatments and bled via wing veins using sterile needles and syringes. 2 mL of blood were collected into properly labelled sterilized bottles containing Ethylenediaminetetra-Acetic Acid (EDTA) as anticoagulant for haematological analysis. The packed cell volume (PCV), red blood cell (RBC), white blood cell (WBC), and the haemoglobin (Hb) concentrations were measured using the Wintrobe's Microhaematocrit, Improved Neubauer Haemocytometer, and Cyanomethaemoglobin methods, respectively [18]. Mean corpuscular haemoglobin (MCH), Mean Corpuscular Volume (MCV), and Mean Corpuscular Haemoglobin Concentration (MCHC) levels were calculated from PCV, Hb, and RBC according to Bush [19].

## 3. Statistical Analysis

 Data collected were subjected to one-way analysis of variance [20], while the differences between the treatment means were compared using Duncan's New Multiple Range Test as outlined by Obi [21]. Statistical significance was assumed at a value of *P* < 0.05. All statistical analysis was performed with SPSS software (version 10.0, SPSS Inc).

## 4. Results and Discussion

 The performance of starter broilers fed *T. occidentalis *leaf extract is as presented in Table 1. There were significant differences (*P* < 0.05) in final body weight, body weight gain, feed conversion, and protein efficiency ratios of birds among the treatments. Birds fed on T_3_ (80 mL FPLE/litre of water) recorded significantly (*P* < 0.05) the highest body weight gain compared to birds in the other treatment groups. There was no significant (*P* < 0.05) variation in weight gain of birds in T_1_ (0 mL), T_2_ (40 mL), T_4_ (120 mL), and T_5_ (160 mL).

The higher weight gain of birds fed 80 mL FPLE suggests that this level may have stimulated higher digestion of the nutrient consumed by the birds and greater efficiency in the utilization of feed resulting in enhanced growth. Kamel [22], Alçiçek et al. [11], and Zhang et al. [12] had earlier reported that plants extracts possess digestion stimulating properties. The result further strengthened the findings of Nworgu [6] and Machebe et al. [3] who reported that broilers tolerated lower levels of *T. occidentalis *and *Gongronema latifolia *leaf extracts, respectively. The comparable weight gain of birds fed higher levels of *T. occidentalis *leaf extracts (120 mL and 180 mL) and the control (0 mL) is an indication that the concentration was within the tolerable limits of the birds and as such did not suppress the growth of the birds. According to Ladeji et al. [15] and Nworgu et al. [23], *T. occidentalis *contains low levels of saponins, tannic acid, phytate, and oxalate which are nutritional factors that depress animal growth. However, in the present study they didnot exert any negative influence on the growth of the animals indicating that the concentration of the toxicants inherent in the concentration of the leaf extract was not toxic to the animals.

There was no significant (*P* < 0.05) variation in the feed intake of birds among the treatment. However, birds fed *T. occidentalis *leaf extract consumed numerically (*P* < 0.05) lower feed than the control. The numerical decrease may be attributed to the improvement in the energy and protein consumption of birds resulting from the enhanced nutrient availability to birds fed *T*. *occidentalis *leaf extracts.

 There was no significant (*P* < 0.05) difference in the water intake of the birds. This implies that *T. occidentalis *did not impact any significant change in the odour and taste of the water, since the sense of taste [24] and smell [25] are important factors in determining what birds consume like other monogastric animals.

 There were significant (*P* < 0.05) differences in the feed conversion and protein efficiency ratios of the birds among the treatments. The feed conversion and protein efficiency ratio of birds fed FPLE were significantly (*P* < 0.05) better than the control while birds fed 80 mL FPLE recorded the best feed conversion and protein efficiency ratios. The best FCR and PER recorded by birds served 80 mL FPLE was as a result of the higher weight gain of the birds. The better FCR observed in birds fed *T. occidentalis *leaf extract suggests that FPLE increased the ability of the birds to utilize available nutrients in the feed and the extract. This observation is in agreement with the reports of Machebe et al. [3] and Nworgu [6] that birds fed leaf extracts had better feed conversion ratio than the control. There was no significant (*P* < 0.05) difference in the PER of birds fed 160 mL/l of *T. occidentalis *leaf extract (T_5_) and the control (T_1_). The enhanced PER observed in birds fed 40 mL/l-120 mL/l of *T. occidentalis *leaf extract is an indication that FPLE positively influenced the protein utilization by the birds.

There was no significant (*P* > 0.05) interactions of the experimental factors with regard to haematological indices among the treatments (Table 2). However, there was marginal increase in the haemoglobin (Hb), Packed Cell Volume (PCV), White Blood Cell (WBC), and Red Blood Cell (RBC) levels of birds fed FPLE. According to Maxwell et al. [26], blood parameters are important in assessing the quality and suitability of feed ingredients for farm animals. Babatunde et al. [27] also reported that blood parameters are the major indices of physiological, pathological, and nutritional status of an organism and changes in the constituent compounds of blood when compared to normal values could be used to interpret the metabolic stage of an animal as well as the quality of feed. The PCV, Hb, RBC, and WBC recorded in the present study fall within the normal range for healthy broiler chicken as reported by Anon [28] and IACUC [29]. This portrays the nutritional status of the birds and thus suggesting adequate nourishment of the birds. It also suggests that the immune system of the birds was adequate. The numerical differences observed in the PCV, Hb, RBC, and WBC of birds fed FPLE inferred that they were better utilized and assimilated into the blood stream for use by the birds [30].

 In conclusion, considering the effects of different levels of *T. occidentalis *leaf extracts on weight gain, feed intake and feed gain ratio of the birds, it appears that 80 mL FPLE/litre of water gave the best result and should be used for broiler starters.

## Figures and Tables

**Table 1:** Performance of starter broiler chicks served *T. occidental *leaves extract supplement.

Parameters	T_1 _	T_2 _	T_3 _	T_4 _	T_5 _	SEM
	0 mL/l	40 mL/l	80 mL/l	120 mL/l	160 mL/l	
Initial body weight (g)	103.89	104.18	106.11	103.21	105.89	3.89
Final body weight (g)	1025.00^b^	1082.78^b^	1200.02^a^	1040.55^b^	990.33^b^	35.60
Body weight gain (g)	921.11^b^	978.60^b^	1093.91^a^	937.34^b^	884.44^b^	29.15
Daily weight gain (g)	26.32^b^	27.96^b^	31.25^a^	26.78^b^	25.27^b^	2.77
Total feed intake (g)	2575.77	2265.39	2258.68	2416.30	2358.83	96.41
Daily feed intake (g)	73.59	64.73	64.53	69.04	67.40	3.16
Total water intake (mL)	4992.09	4625.89	4624.38	4715.51	4529.85	129.85
Daily water intake (mL)	142.63	132.17	132.13	134.73	129.42	5.72
Feed conversion Ratio	2.80^c^	2.32^b^	2.06^a^	2.58^b^	2.67^b^	0.19
Protein efficiency	1.70^c^	1.98^b^	2.46^a^	1.85^b^	1.78^bc^	0.35

^abc^ Means with different superscripts on the same row differ significantly (*P* < 0.05).

**Table 2:** Haematological parameters of starter broilers served *T. occidentalis* leaves extract supplement.

Parameters	T_1 _	T_2 _	T_3 _	T_4 _	T_5 _	SEM
	0 mL/l	40 mL/l	80 mL/l	120 mL/l	40 mL/l	
Haemoglobin (g/L)	11.00	12.75	11.80	11.50	11.10	1.32
Packed cell volume (%)	33.50	38.00	34.00	34.50	33.95	4.10
Red blood cell (× 10^9^/L)	2.63	2.75	2.86	2.85	2.83	1.01
White blood cell (×10^6^/L)	2.29	2.48	2.55	2.44	2.40	1.62
Mean corpuscular volume (f1)	4.15	3.90	4.00	3.80	3.70	0.24
Mean cell haemoglobin (pg)	1.35	1.31	1.33	1.26	1.23	0.05
Mean cell haemoglobin concentration (g/L)	328.35	335.42	337.50	346.36	331.37	10.56
